# A direct comparison of CellSearch and ISET for circulating tumour-cell detection in patients with metastatic carcinomas

**DOI:** 10.1038/bjc.2011.294

**Published:** 2011-08-09

**Authors:** F Farace, C Massard, N Vimond, F Drusch, N Jacques, F Billiot, A Laplanche, A Chauchereau, L Lacroix, D Planchard, S Le Moulec, F André, K Fizazi, J C Soria, P Vielh

**Affiliations:** 1 Translational Research Laboratory, Institut de Cancérologie Gustave Roussy, 94805 Villejuif, France; 2 University of Paris-Sud, INSERM U 981 ‘Identification of molecular predictors and new targets for cancer treatment’, Institut de Cancérologie Gustave Roussy, 94805 Villejuif, France; 3 Department of Medicine, Institut de Cancérologie Gustave Roussy, 94805 Villejuif, France; 4 Department of Biostatistics, Institut de Cancérologie Gustave Roussy, 94805 Villejuif, France; 5 Department of Oncology, Hôpital d’Instruction des Armées du Val de Grace, 75230 Paris, France; 6 Department of Biopathology, Institut de Cancérologie Gustave Roussy, 94805 Villejuif, France

**Keywords:** circulating tumour cell, breast cancer, prostate cancer, lung cancer, CellSearch, ISET

## Abstract

**Background:**

Circulating tumour cells (CTCs) can provide information on patient prognosis and treatment efficacy. However, there is no universal method to detect CTC currently available. Here, we compared the performance of two CTC detection systems based on the expression of the EpCAM antigen (CellSearch assay) or on cell size (ISET assay).

**Methods:**

Circulating tumour cells were enumerated in 60 patients with metastatic carcinomas of breast, prostate and lung origins using CellSearch according to the manufacturer's protocol and ISET by studying cytomorphology and immunolabelling with anti-cytokeratin or lineage-specific antibodies.

**Results:**

Concordant results were obtained in 55% (11 out of 20) of the patients with breast cancer, in 60% (12 out of 20) of the patients with prostate cancer and in only 20% (4 out of 20) of lung cancer patients.

**Conclusion:**

Our results highlight important discrepancies between the numbers of CTC enumerated by both techniques. These differences depend mostly on the tumour type. These results suggest that technologies limiting CTC capture to EpCAM-positive cells, may present important limitations, especially in patients with metastatic lung carcinoma.

Metastatic dissemination of malignant solid tumours is the main cause of death by cancer in the developed countries. The metastatic cascade is defined as a series of biological events that cancer cells from the primary neoplasia must complete to develop a new malignancy at a distant site, including the crucial step consisting in the release and survival of tumour cells in the peripheral blood (Sahai, 2007). These circulating tumour cells (CTCs) consist in a heterogeneous population of very rare cells native of either the primary tumour or its own metastasis (Mocellin *et al*, 2006; Sahai, 2007; Alix-Panabières *et al*, 2008). Key molecular events involved in metastatic progression are likely to have considerable effects on the number and on the phenotype of CTC. In this regard, experimental data suggest that biological processes such as the epithelial-to-mesenchymal transition (EMT), associated with the acquisition of cancer stem cell properties and resistance to conventional therapy, might have a role in the generation of more aggressive sub-populations of CTC (Polyak and Weinberg, 2009; Mego *et al*, 2010). Although the existence of CTC has been known for over a hundred of years, only the recent advent of novel cytometric (i.e., whole cell based) technologies have enabled significant progress in detecting and quantifying these cells. Using the CellSearch platform (Veridex, Warren, NJ, USA), several groups have shown an association between the counts of CTC identified by the assay and patients’ clinical outcome (Cristofanilli *et al*, 2004; Cohen *et al*, 2008; De Bono *et al*, 2008). Circulating tumour cell levels were indeed prospectively demonstrated to be an independent prognostic factor in patients with advanced breast, prostate and colorectal cancers treated by conventional and/or hormonal therapy (Cristofanilli *et al*, 2004; Cohen *et al*, 2008; De Bono *et al*, 2008). Moreover, longitudinal monitoring also indicated a potential role for enumerating CTC as an endpoint biomarker making it possible to predict, at an early stage, whether a patient benefits or not from anticancer treatment (Hayes *et al*, 2006). In addition to prognostic and predictive utility, CTC also represent a unique non-invasive source of tumour material susceptible to provide real-time information on the patient's current disease status. New possibilities with important clinical implications therefore concern the molecular characterisation of CTC since it could be incorporated in future clinical designs to monitor the efficacy of or resistance to targeted therapy.

Numerous technical efforts have been made to reliably detect and quantify CTC, but the development of a universal assay has proven quite difficult. Circulating tumour cells are indeed very rare events, occurring at rates as low as one cell per 10^6^ or 10^7^ leukocytes and most methods rely on the combination of two steps, that is, enrichment followed by detection to increase the sensitivity of the assay (Mostert *et al*, 2009). Another major technical challenge concerns tumour heterogeneity. Gene-expression profiling has highlighted the remarkable heterogeneity of malignant cells not only within a given histological subtype but also among tumour cells of any given patient. In addition to genetic instability inherent to most neoplastic cell types, emerging data suggest that cell-biological changes during metastatic progression, such as the transition between epithelial-to-mesenchymal states, can also generate distinct multiple cellular sub-populations contributing to intratumoural heterogeneity (Polyak and Weinberg, 2009; Thiery *et al*, 2009; Mego *et al*, 2010). Owing to this heterogeneity, the assay used for CTC detection could strongly impact on the biomarker value of CTC, and data regarding CTC should therefore be interpreted in the context of this method.

A rather limited number of studies comparing the performance of different assays and providing data to support the choice of a given method have been published (Lambrechts *et al*, 1999; Smith *et al*, 2000; Ring *et al*, 2005; Van der Auwera *et al*, 2010). This study was designed to directly compare the performance of two cytometric (i.e., CellSearch and ISET) systems based on different CTC properties, namely the expression of an epithelial antigen membrane *vs* cell size. The standardised and semi-automated CellSearch platform is the only assay currently approved by the Food and Drug Administration. Circulating tumour cell enrichment by CellSearch is based on the expression of the epithelial-lineage marker EpCAM (epithelial cell adhesion molecule). EpCAM-positive cells are enriched by immunomagnetic separation using EpCAM-specific antibodies conjugated to magnetic particles and then stained with fluorescent anti-cytokeratin and 4′,6-diamino-2-phenylindole (DAPI), while hematopoietic cells are stained with anti-CD45 antibodies. Cytokeratin and DAPI-positive, and CD45-negative CTC are finally counted by using a semi-automated fluorescent microscope (Cristofanilli *et al*, 2004; Hayes *et al*, 2006; Cohen *et al*, 2008; De Bono *et al*, 2008). ISET (isolation by size of epithelial tumour cells) makes it possible to collect tumour cells based on their larger size as cells are enriched by blood filtration through filtering membranes with calibrated pores 8 *μ*m in diameter (Vona *et al*, 2000, 2004; Paterlini-Brechot and Benali, 2007). Enriched cells are stained on the filter for cytomorphological examination or further characterised by immunocytochemistry. As the performance of CTC detection assays may also be influenced by intrinsic characteristics of different tumour types, this study comprises 60 patients with metastatic carcinomas of breast, prostate and lung origins.

## Patients and methods

### Blood sample collection

This study was approved by our institutional review board and local ethics committee. Informed and written consent was obtained from all patients. Peripheral blood samples were collected from 60 patients with histologically or cytologically confirmed metastatic breast (*n*=20), prostate (*n*=20) or non-small cell lung cancer (*n*=20). Clinico-pathologic information was recorded for all patients. For each patient, 10 ml of blood was collected in EDTA tubes (Terumo, Leuven, Belgium) for CTC enumeration by ISET and 7.5 ml was collected in CellSave collection tubes (Immunicon Inc., Huntingdon Valley, PA, USA) for the CellSearch test.

### Enumeration of CTC by CellSearch

Circulating tumour cell enumeration using the CellSearch system (Veridex LLC, Raritan, NJ, USA) was carried out according to the manufacturer's protocol and training. Blood samples were processed on the CellTracks Autoprep within 72 h. Epithelial cells were immunomagnetically enriched using ferrofluids coated with EpCAM-specific antibodies, then permeabilised and fluorescently labelled with phycoerythrin-conjugated antibodies directed against cytokeratin 8, 18 and 19, an allophycocyanin-conjugated antibody to CD45 and the nuclear dye DAPI. After transfer to a cartridge in a MagNest, labelled cells were analysed on the CellTracks Analyser II, a four-colour semi-automated fluorescent microscope that captures images of the entire surface of the cartridge for the four fluorescent dyes. From the captured images, a gallery of objects was presented to a trained operator (technician) who interprets each object. According to manufacturer guidelines, an object defined as a CTC should meet all of the following criteria: (i) an intact cell with a round to oval morphology and at least 4 *μ*m in size; (ii) positive for DAPI with a nucleus inside the cytoplasm (of at least >50%) and a nucleus area smaller than the cytoplasm; (iii) positive for cytokeratins (bright or moderate) and negative for CD45 and the blank channel. Each sample was analysed independently by two trained technicians (NV and NJ).

### Enumeration of CTC by ISET

ISET was carried out as previously reported (Vona *et al*, 2000, 2004; Paterlini-Brechot and Benali, 2007). Blood samples (10 ml) were processed within 4 h, diluted 1 : 10 in an erythrocyte-lysis buffer and filtered on the ISET device. The module filtration had a membrane of 10 wells making it possible to process blood samples of 10 ml (i.e., 100 ml of diluted blood). After filtration, membranes were washed with PBS, disassembled from the filtration module, allowed to air-dry overnight and stored at −20 °C until staining. Before staining, membranes were thawed, hydrated in TBS (Dako, Glostrup, Denmark), and treated at 98 °C in EDTA pH: 9.9, for 20 min, for antigenic retrieval. After being rinsed in TBS and dried at room temperature, membranes were permeabilised 5 min in the presence of TBS containing 0.2% Triton X100 (Roche, Mannheim, Germany) and incubated in methanol containing 3% H_2_0_2_ to block endogenous peroxydase. Membranes of patients with breast or lung cancer were incubated overnight at 4 °C in wet chambers, with an antibody raised against cytokeratin 7 (Dako), a keratin usually expressed in both malignant breast and lung tumours whereas those of patients with prostate cancer were stained with an anti-p504S antibody (Dako) directed to alpha-methylacyl coenzyme A racemase, a marker specifically and highly expressed in prostate adenocarcinoma. After rinsing in TBS, membranes were incubated with a secondary antibody labelled with peroxydase (Dako) for 45 min at room temperature. Finally, membranes were rinsed again in TBS, treated with diaminobenzidine (Dako) for 10 min and counterstained. Each immunostaining experiment included positive (a positive cell line stained with the specific antibody) and negative (the positive cell line stained with a nonspecific antibody of the same isotype and at the same concentration than the specific antibody) controls. Stained membranes were examined by a trained technician using light microscopy in two steps: (i) screening at × 20 magnification to locate cells, (ii) observation at × 63 magnification with oil immersion for detailed cytomorphological analysis. Isolated and/or clusters of cells of interest (immunostained or not) were selected, digitised, and examined by an experienced cytopathologist (PV). Circulating tumour cells were defined as cells presenting all the following criteria: (i) nuclear size equal or larger than two pores (i.e., equal or larger than 16 *μ*M); (ii) irregularity of the nuclear contour; (iii) presence of a visible cytoplasm; and (iv) high nuclear-to-cytoplasmic ratio (>0.8). When one or several of the above criteria were missing, cells were classified as atypical. Samples of 10 ml were usually processed on ISET, and calculations were made in order to express CTC in 7.5 ml.

### Statistical methods

As CTC levels in patients were not normally distributed, results were presented as counts and medians with the corresponding percentages and ranges. Linear regression plots were computed for CTC counts obtained by the CellSearch and ISET techniques. As a result of the low number of patients and of CTC count distribution, CTC values determined using both CellSearch and ISET technique were correlated by the Spearman test.

## Results

**Figure 1:**
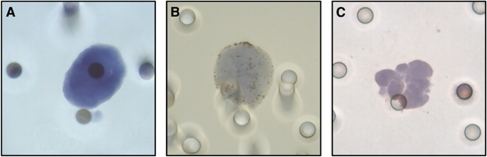
Microscopic analysis of CTC by ISET. (**A**–**C)** Examples of isolated CTC (**A**, **B**) and of a cluster of CTC (**C**) detected in a patient with metastatic prostate adenocarcinoma. Circulating tumour cells were enriched using the ISET device and stained with the anti-p504S monoclonal antibody. Circulating tumour cells were identified according to the following cytomorphological criteria: (i) nuclear size equal to or larger than two pores (i.e., equal to or larger than 16 *μ*M); (ii) the irregularity of the nuclear contour; (iii) the presence of a visible cytoplasm; (iv) a high nuclear-to-cytoplasmic ratio (>0.8). The 8 *μ*m width pores are visible on (**A**–**C**).

Each of the 60 patients with metastatic breast (MBC), prostate (MPC) and lung (MLC) carcinomas had blood collected for CTC enumeration by both the CellSearch and ISET techniques. Tables 1, 2 and 3 respectively show the clinico-pathological characteristics of the patients with MBC (*n*=20), MPC (*n*=20) and MLC (*n*=20), as well as their respective CTC counts obtained by both techniques. Representative examples of isolated CTC (panels A and B) and of a CTC cluster (panel C) detected by ISET are shown in Figure 1.

**Figure 2:**
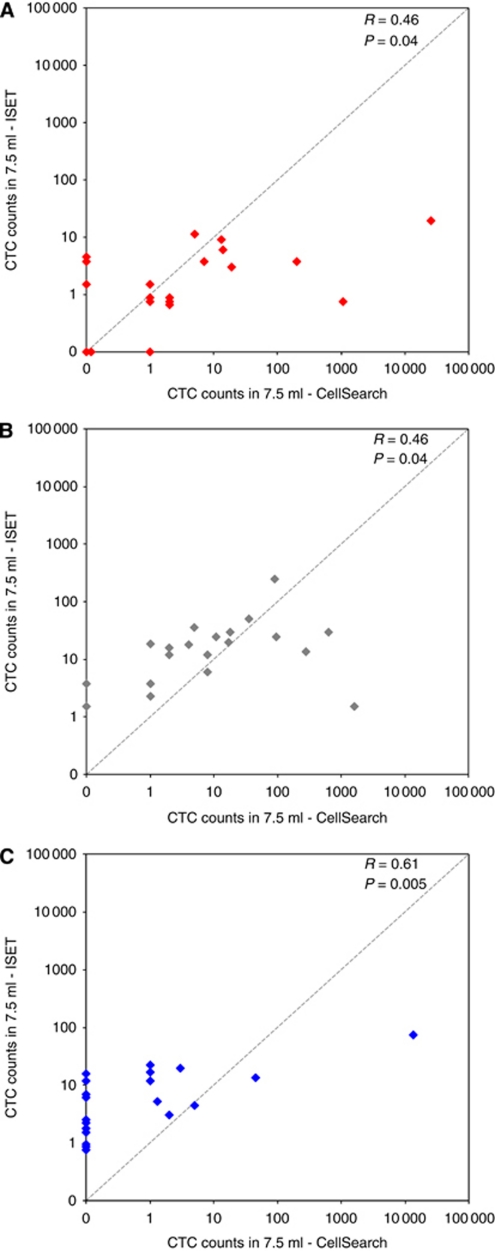
Circulating tumour cells counts by CellSearch and ISET in patients with metastatic carcinomas of breast (MBC), prostate (MPC) and of lung (MLC) origin. (**A**) Circulating tumour cell counts by CellSearch and ISET in patients with MBC. (**B**) Circulating tumour cell counts by CellSearch and ISET in patients with MPC. (**C**) Circulating tumour cell counts by CellSearch and ISET in patients with MLC.

**Figure 3:**
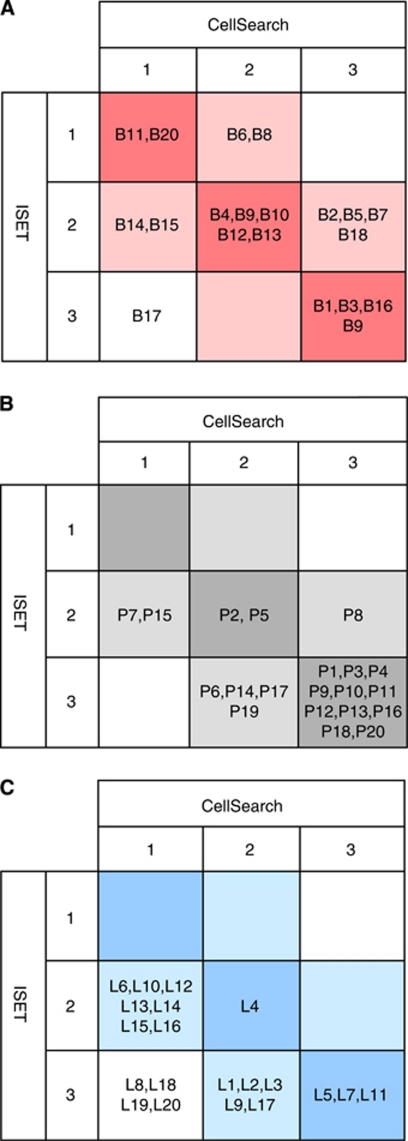
Classification of patients according to the numbers of CTC detected by CellSearch and ISET. (**A**) Classification of MBC patients according to the numbers of CTC detected by CellSearch and ISET. (**B**) Classification of MPC patients according to the numbers of CTC detected by CellSearch and ISET. (**C**) Classification of MLC patients according to the numbers of CTC detected by CellSearch and ISET. For each technique, patients with no detectable CTC were classified in group 1, patients with CTC levels ranging from 1 to 4 CTC/7.5 ml were classified in group 2 and patients with CTC levels equal or superior to 5 CTC/7.5 ml were classified in group 3.

The median CTC count was of 2 CTC/7.5 ml for both techniques (range, 0–25 500 for CellSearch, range, 0–20 for ISET) in MBC patients. Circulating tumour cell counts for the two methods obtained were weakly correlated (*r*=0.46, *P*=0.04) (Figure 2A). Of the 20 patients with MBC, eight (40%) had CTC counts equivalent or superior to 5/7.5 ml by CellSearch while five (20%) had CTC counts ⩾5/7.5 ml by ISET (Table 1). Among the five negative patients using CellSearch, three were found positive by ISET, whereas of the three patients negative using ISET, one patient was found positive by CellSearch. Only two (10%) patients were negative using both techniques while discordant findings were obtained in four (15%) patients (Figure 2A). In patients with CTC detectable by both techniques, CTC counts were generally higher using CellSearch than by ISET. To further examine the concordance of both techniques, patients were classified according to the numbers of CTC detected by each technique: (i) patients with no detectable CTC (group 1); (ii) patients with CTC levels ranging from 1 to 4 CTC/7.5 ml (group 2); and (iii) patients with CTC levels equal or superior to 5 CTC/7.5 ml (group 3). Data are shown in Figure 3A. Eleven patients were found in the same group indicating a good concordance between both techniques in approximately half of the patients. In contrast, six patients had higher CTC counts using CellSearch than by ISET, while three patients had CTC counts higher using ISET than by CellSearch. Of the five patients with triple-negative breast cancer for ER, PR and HER2, a phenotype that has been reported to express cancer-stem cell and mesenchymal markers, and exhibit a more aggressive phenotype possibly associated with the EMT process and the downregulation of epithelial markers (Sarrió *et al*, 2008; Qi *et al*, 2010), two patients (B1, B5) had higher CTC counts using CellSearch than those obtained by ISET. Therefore, in contrast to a recent report using tumour cell lines spiked in normal blood samples (Sieuwerts *et al*, 2009), our study suggests that the capacity of the CellSearch system does not fail to detect CTCs in such patients.

In MPC patients, the median CTC count was of 8 CTC/7.5 ml (range, 0–1621) for CellSearch) and 17 CTC/7.5 ml (range, 1–248) for ISET. Circulating tumour cell counts for the two methods were again weakly correlated (*r*=0.46, *P*=0.04) (Figure 2B). Of the 20 MPC patients, 12 (60%) had CTC counts ⩾5/7.5 ml according to CellSearch while 15 (75%) had CTC counts ⩾5/7.5 ml using ISET (Table 2). All patients had detectable CTC by ISET while two patients were negative by CellSearch (P7 and P15 with 4 and 2 CTC/7.5 ml by ISET, respectively) (Table 2, Figure 2B). According to the classification already depicted, Figure 3B shows that 13 patients were classified in the same group indicating that concordant results were observed in 60% of the patients. Furthermore, six patients (30%) had CTC counts markedly higher according to ISET than CellSearch. Only one patient (P8) had 1 CTC/7.5 ml using ISET and 1621 CTC/7.5 ml according to CellSearch. No relationship was observed between clinico-pathological characteristics of these patients and their respective counts of CTC measured by each technique.

The median CTC count was of 0 CTC/7.5 ml (range, 0–13 500) for CellSearch and 5 CTC/7.5 ml (range, 1–100) for ISET in MLC patients. Circulating tumour cell counts obtained with both methods were moderately correlated (*r*=0.61, *P*=0.005) (Figure 2C). Of the 20 patients with MLC, 3 (15%) had CTC counts ⩾5/7.5 ml according to CellSearch, while 12 (60%) had CTC counts ⩾5/7.5 ml using ISET (Table 3). Eleven (55%) patients were negative according to CellSearch, while all patients were positive using ISET. Figure 3C shows that concordant results were obtained in only 4 patients (20%) while 16 (80%) patients had CTC counts markedly higher with ISET than CellSearch. In particular, four patients had CTC counts ⩾5/7.5 ml according to ISET and were negative using CellSearch. In one patient (L11), CTC counts were higher with CellSearch than with ISET (46 *vs* 14 CTCs/7.5 ml).

## Discussion

Our prospective and comparative study of 60 patients with metastatic carcinoma demonstrates quite considerable discrepancies between the number of CTC enumerated by the CellSearch and the ISET systems. In all, 30% of patients (18 out of 60) were negative according to CellSearch while only 5% (3 out of 60) were negative using ISET. Concordant results only concern 28 of 60 patients (47%), whereas discordant results consist of patients with CTC counts higher according to ISET (25 out of 60; 42%) or with CTC counts higher using CellSearch (7 out of 60; 12%). Interestingly, these differences mostly depend on the type of tumour that the patient is harbouring.

In patients with MBC, CTC counts are generally higher by CellSearch than by ISET. The lower detection using ISET may be explained by the loss of CTC at different times of the process: (i) through the pores of 8 *μ*M during the procedure of filtration; (ii) during antigenic retrieval performed at 98 °C in an alkaline (pH: 9.9) buffer before immunolabelling, and/or; (iii) during the sequential washes used during the immunostaining procedure performed after filtration. Alternatively, CTC identified by CellSearch may not be true CTC. Indeed, CTC are detected by CellSearch on the basis of the expression of an epithelial marker (EpCAM), which does not formally establish the malignant nature of circulating cells in the blood retained as CTC. However, the specificity of CellSearch has been reliably documented in normal individuals and in patients with benign tumours (Allard *et al*, 2004). Therefore, the lower CTC counts obtained by ISET compared with CellSearch, likely results from cell loss during the ISET procedure. In order to minimise this cell loss, we currently bypass the critical antigen retrieval step and use more sensitive methods with immunofluorescent antibodies.

Overall, our results indicate a better detection of CTC in patients with MPC and MLC, *via* ISET than CellSearch. Low count of CTC with CellSearch has already been reported by other groups in MLC (Allard *et al* 2004; Okumura *et al* 2009). However, this study provides direct evidence for the first time that CTCs are underestimated by CellSearch in MPC and MLC patients because higher CTC levels are detected using another technique. All patients with MPC and 17 out of 20 patients with MLC had primary carcinomas of glandular origin, usually expressing the EpCAM antigen. These results may, in part, reflect data observed in experimental tumour models suggesting a continuum during the so-called EMT with the development of discrete tumour phenotypes, ranging from epithelial differentiation to mesenchymal phenotype and including patterns with various epithelio–mesenchymal mixed phenotypes (Mego *et al*, 2010). As tumour cells undergoing the EMT process are mainly characterised by the loss of epithelial markers, the neoexpression of cytoplasmic mesenchymal markers and of additional markers not detectable by CellSearch, the ISET system may be much more efficient in identifying all the cells of interest involved in the process. This has been recently shown in a series of patients with resectable lung cancer, where a significant proportion of CTC identified by ISET either co-expressed cytokeratins and vimentin or expressed vimentin alone (Hofman *et al*, 2011a). In this context, the use of alternative morphology-based enrichment technique such as ISET may offer significant advantages. However, this assay is still cumbersome, time consuming, and despite recent efforts (Hofman *et al*, 2011b) not standardised enough in its current form to be routinely applicable in clinical studies.

The criteria used by CellSearch for identifying CTC are mainly based on the size of the cytokeratin fluorescent signal that should be superior to 4 *μ*m and on the location of the DAPI signal, which should be at least 50% inside the cytokeratin signal. It is noteworthy that CTC detection by CellSearch does not rely on any true morphological criteria. In contrast, CTC detected by ISET were identified by an experienced cytopathologist (PV) according to basic morphologic criteria such as a nucleus with a size equal to or larger than two pores (16 *μ*m), irregularity of the nuclear contour, the presence of a visible cytoplasm and a high nuclear-to-cytoplasmic ratio (>0.8). The size of a normal eukaryotic cell usually ranges from 8 to 100 *μ*m and tumour cells are generally bigger than their normal counterpart. The threshold of 16 *μ*m (two-fold the diameter of a filter pore and the size of the smallest normal cell) was set to exclude most of the normal circulating blood cells. Several cells were classified as atypical because they either presented some but not all cytomorphological criteria (for example, nucleus size smaller than 16 *μ*m) or were damaged. We obviously assume that some of these atypical cells may be malignant leading to an underestimation of CTC counts by ISET. Future studies combining morphological, phenotypical and molecular characterisation will provide additional information assessing or not their tumoural origin. We also decided to retain as CTC cells those that presented all the expected cytomorphological criteria required to establish the diagnosis of tumour cells, even if they were not stained by specific antibodies (data not shown). As we used an aggressive staining procedure based on antigenic retrieval to improve immunostaining, it is possible that some of these unstained cells were CTC negative for the selected marker thus potentially reflecting tumour cell phenotypic heterogeneity. Microemboli (namely, clusters of CTCs) that are rarely detectable by CellSearch were frequently observed by ISET (Figure 1). Within these microemboli, CTC counting was quite difficult and may have been underestimated in some patients.

In conclusion, by directly comparing CellSearch and a morphology-based enrichment technique (ISET), our study provides strong evidence that the CellSearch system, as well as potentially other current technologies that limit CTC capture to EpCAM-positive cells, does present important limitations. The limitations of the CellSearch system mainly concern patients with MLC, supporting the hypothesis of a phenotypic heterogeneity possibly linked to downregulation of the epithelial phenotype in these patients. Whether ISET is a more appropriate technique to enumerate and characterise CTC in patients bearing certain types of metastatic tumours such as MLC, is still a matter of debate. Our study did not compare the clinical relevance of both methods. Further studies on larger cohorts of patients are obviously needed to assess this important issue. ISET could indeed represent a more accurate clinical tool for predicting patient's outcome in certain tumour types, and provide a significant advantage for performing molecular analyses in the era of personalised medicine.

## Figures and Tables

**Table 1:** Clinico-pathological characteristics of patients with metastatic breast cancer with their respective CTC counts

					**ISET**		**CellSearch**
**Patients** a	**Age** b	**Histology**	**ER/PR/HER2 status**	**TNM stage**	**CTC/7.5 ml**	**Atypical cells/7.5 ml**	**CTC/7.5 ml**
B1	56	IDC	ER^−^PR^−^HER2^−^	T2N0M0	6	25	14
B2	49	IDC	ER^+^PR^+^HER2^−^	T3N1M0	4	14	200
B3	56	ILC	ER^+^PR^+^HER2^−^	T3N2M0	20	165	25 500
B4	39	IDC	ER^+^PR^+^HER2^−^	TxNxM1	1	4	2
B5	36	IDC^c^	ER^−^PR^−^HER2^−^	T3N2M0	3	6	19
B6	61	IDC	ER^+^PR^−^HER2^+^	T4dN2Mx	1	0	2
B7	49	IDC	ER^−^PR^−^HER2^+^	T4dN1M0	1	1	1078
B8	70	IDC	ER^−^PR^−^HER2^+^	T2N1M0	0	1	1
B9	43	IDC	ER^−^PR^−^HER2^+^	T2NxM1	2	2	1
B10	68	IDC	ER^+^HER2^+^	TxN-M0	1	7	1
B11	38	IDC	ER^−^PR^−^HER2^−^	T2N0M0	0	2	0
B12	68	IDC	ER^+^PR^+^HER2^−^	T1cN1M0	1	12	1
B13	43	IDC^d^	ER^+^PR^+^HER2^+^	T2N0Mx	1	5	2
B14	49	IDC	ER^−^PR^−^HER2^−^	T2N0M0	4	17	0
B15	76	IDC	ER^−^PR^−^HER2^−^	T4dN0M0	2	18	0
B16	61	IDC	ER^+^PR^−^HER2^−^	T2N0Mx	9	13	13
B17	61	IDC	ER^+^PR^−^	T2N1M0	5	13	0
B18	56	IDC	ER^+^	T2N1M0	4	25	7
B19	72	IDC	ER^+^PR^+^HER2^−^	T4bN1M1	11	26	5
B20	41	IDC	ER^+^PR^+^HER2^−^	T3N1M0	0	2	0

Abbreviations: CTC=circulating tumour cell; ER=oestrogen receptor; F=female; HER2=human epidermal growth factor receptor 2; IDC=infiltrative ductal carcinoma; ILC=infiltrative lobular carcinoma; PR=progesterone receptor; TNM=tumour node metastasis.

a All patients were female.

b Age (years) at the moment of CTC analysis.

c Infiltrative mucinous carcinoma.

d Infiltrative apocrine carcinoma.

For patients B10, B17 and B18, PR, HER2 and PR and HER2 were not available, respectively.

**Table 2:** Clinico-pathological characteristics of patients with metastatic prostate cancer with their respective CTC counts

						**ISET**		**CellSearch**
**Patients**	**Age** a	**Histology**	**Gleason grade**	**TNM stage**	**PSA (*μ*g l^–1^)** b	**CTC/7.5 ml**	**Atypical cells/7.5 ml**	**CTC/7.5 ml**
P1	80	IA	8	T2N0M1	196	12	5	8
P2	61	IA	6	T2N0M1	14.6	2	5	1
P3	75	IA	7	T3N0M1	1095	248	0	90
P4	66	IA	ND	T2N0M1	1394	30	120	643
P5	73	IA	9	T2N0M1	603	4	35	1
P6	73	IA	9	T2N0M1	60	12	55	2
P7	82	IA	8	T3N0M1	97	4	13	0
P8	62	IA	7	T3N0M1	2669	1	18	16 121
P9	70	IA	8	T2N1M1	68	25	16	11
P10	78	IA	8	T2N0M0	354	20	59	17
P11	88	IA	8	T3N0M1	77	30	30	18
P12	50	IA	9	T1N0M0	101	6	18	8
P13	50	IA	9	T3N0M1	93	35	18	5
P14	67	IA	7	T2N0M1	1.8	16	15	2
P15	67	IA	9	T3dN0M1	3.8	2	9	0
P16	83	IA	7	T2N0M1	689	14	43	278
P17	78	IA	10	T4N0M1	50	19	24	1
P18	82	IA	9	T2N0M1	85	25	43	95
P19	68	IA	9	T3N2M0	0.2	18	68	4
P20	80	IA	8	T3N1M0	40	50	185	36

Abbreviations: CTC=circulating tumour cell; IA=infiltrative adenocarcinoma; ND=not determined; PSA=prostate serum antigen; TNM=tumour node metastasis.

a Age (years) at the moment of CTC analysis.

b PSA at the moment of CTC analysis.

**Table 3:** Clinico-pathological characteristics of patients with metastatic lung cancer with their respective CTC counts

					**ISET**		**CellSearch**
**Patients**	**Age** a	**Sex**	**Histology**	**TNM stage**	**CTC/7.5 ml**	**Atypical cells/7.5 ml**	**C/7.5 ml**
L1	61	M	IA^b^	pT2N0M1	23	7	1
L2	63	F	SC	pT2N2M1	20	28	3
L3	38	F	EC	pT3N3M1	12	3	1
L4	56	F	IA	T3N3M1	3	4	2
L5	59	M	IA	T4N3M1	5	9	5
L6	56	M	IA	T3N2M1	2	2	0
L7	55	F	IA^b^	T2N3M1	>100	0	13 500
L8	72	M	NE	pT3N0M1	16	10	0
L9	67	F	IA	pT2N2M1	5	22	1
L10	60	M	IA	T3N3M1	2	2	0
L11	60	M	IA	T2N2M1	14	7	46
L12	51	F	IA	pT2N0M1	2	2	0
L13	56	M	IA	T2N2M1	1	7	0
L14	61	F	IA	pT2N0M1	2	9	0
L15	38	M	IA	T4N2M1	1	2	0
L16	54	M	EC	pT4N2M1	1	0	0
L17	51	F	IA	T2N2M1	17	28	1
L18	61	M	IA	T2N2M1	12	53	0
L19	42	M	IA^c^	T2N0M?	6	5	0
L20	54	F	IA	pT2N2M0	7	46	0

Abbreviations: CTC=circulating tumour cell; EC=infiltrative epidermoid carcinoma; F=female; IA=infiltrative adenocarcinoma; M=male; NE=large cell neuroendocrine carcinoma; SC=sarcomatoid carcinoma with giant cells; TNM=tumour node metastasis.

a Age (years) at the moment of CTC analysis.

b Poorly differentiated.

c Bronchoalveolar.
